# Supplementary material for: Potential Therapeutic Skin Microbiomes Suppressing Staphylococcus aureus-Derived Immune Responses and Upregulating Skin Barrier Function-Related Genes via the AhR Signaling Pathway
Source: Int J Mol Sci. 2022 Aug 23;23(17):9551. doi: 10.3390/ijms23179551 (PMC9455615; doi:10.3390/ijms23179551)
Supplement: Supplementary file 1 [file ijms-23-09551-s001.zip › ijms-1880922-supplementary.pdf]

Supplementary Material

Potential therapeutic skin microbiomes suppressing Staphylococcus aureus-derived immune response and upregulating skin barrier function related genes via AhR signaling pathway

Eulgi Lee[1], Kyungchan Min [1], Hyeok Ahn [1], Bu-nam Jeon [2], Shinyoung Park [2], Changhee Yun [2], Hyehee Jeon [2], Jae-sung Yeon [2], Hyun Kim [1] and Hansoo Park [1,2]*

[1] Department of Biomedical Science and Engineering, Gwangju Institute of Science and Technology (GIST), Gwangju 61005, Republic of Korea
[2] Genome and Company, Pangyo-ro 255, Bundang-gu, Seoungnam-si, Gyeonggi-do 13486, Republic of Korea
* Correspondence: hspark27@gist.ac.kr

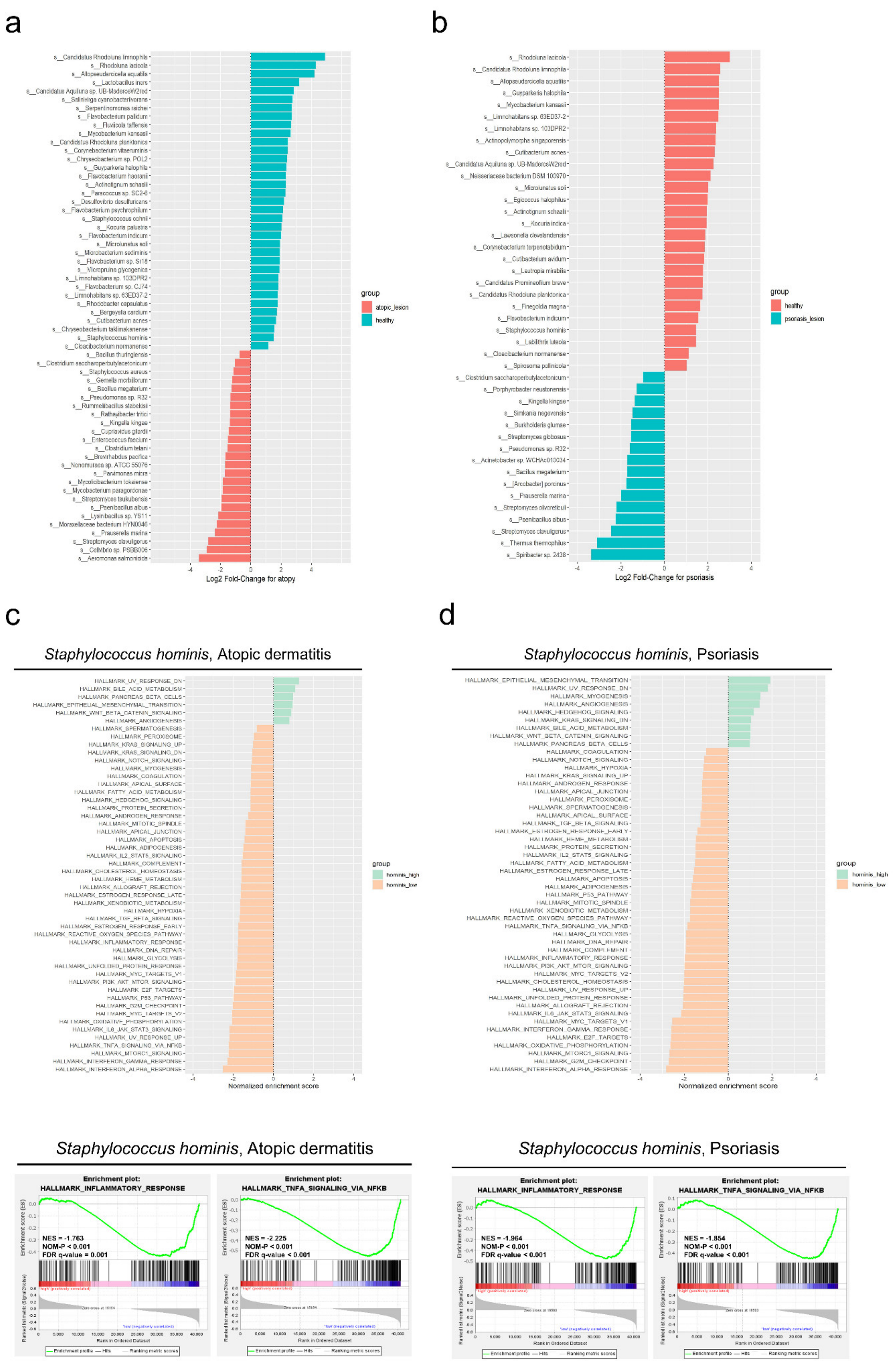

Figure S1. Identification of functional microbiomes in atopic dermatitis (AD) and psoraissi (Pso) cohorts and modulation of pathogenesis based on differences in abundance of *Staphylococcus hominis*. Linear discriminant

analysis (LDA) effect sizes (LEfSe) for two populations: **(a)** healthy controls versus AD lesions and **(b)** healthy controls versus Pso lesions. As indicated by the vertical dots, the length of the bar represents the $\log_2$ transformed LDA score. Alongside the horizontal lines are the taxa of bacteria whose relative abundance change is statistically significant ($p < 0.05$). **(c)** Normalized enrichment scores (NES) of Gene Set Enrichment Analysis (GSEA)-based gene sets according to *S. hominis* abundance in the AD cohort. Below that, GSEA according to *S. hominis* abundance in the AD cohort ([NOM] P < 0.001; inflammatory response, [NOM] P < 0.001; TNF-α signaling via NFκB). (**d**) Normalized Enrichment Scores (NES) of GSEA-based gene sets according to *S. hominis* abundance in the Pso cohort. Below that, GSEA according to *S. hominis* abundance in the Pso cohort ([NOM] P < 0.001; inflammatory response, [NOM] P < 0.001; TNF-α signaling via NFκB).

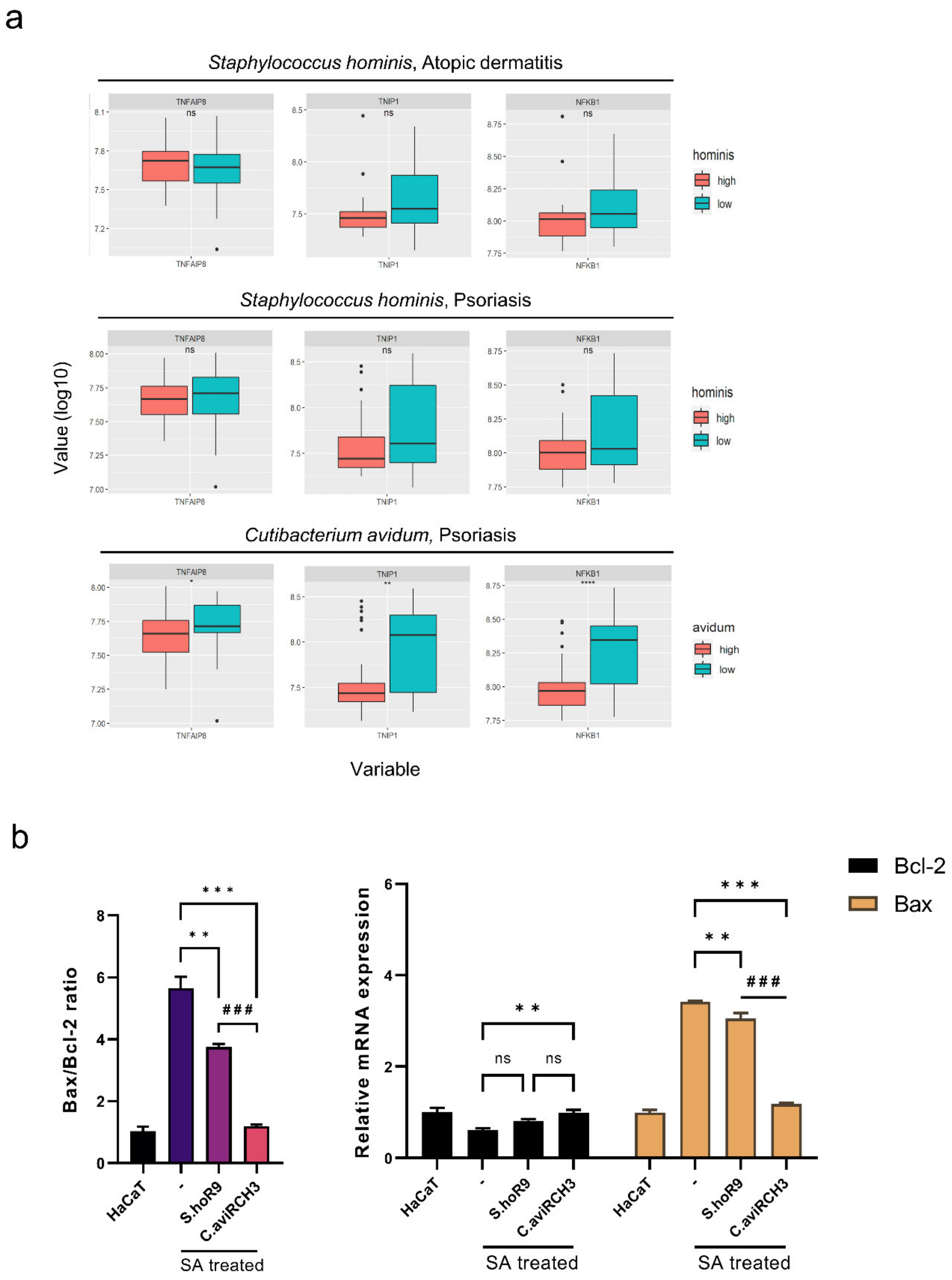

Figure S2. An integrated functional microbiome suppresses apoptosis-related genes while reducing mitochondrial apoptosis derived from ROS markers. **(a)** Boxplots of gene expression values for *TNFAIP8*, *TNP1* and *NFκB1* based

on abundance of *S. hominis* in AD and Pso cohorts. **(b)** Measurement of the *Bax*/*Bcl-2* ratio for mitochondrial apoptosis induced by *Staphylococcus aureus* (SA) and by SA treatment in combination with *Cutibacterium avidum* G01 and *S.hominis* (r9). Next to that, measurement of mRNA levels of *Bax* and *Bcl-2* relative to 18S rRNA after SA treatment and by SA treatment in combination with *C. avidum* G01 and *S. hominis* (r9) **p* < 0.05, ***p* < 0.01, ****p* < 0.001, ns: not-significant compared to SA only treatment group. ###*p* < 0.001, ns: not-significant compared between SA with *S. hominis* R9 and SA with *C. avidum* G01 treatment groups. Statistical significance was calculated using Bonferroni tests.

a

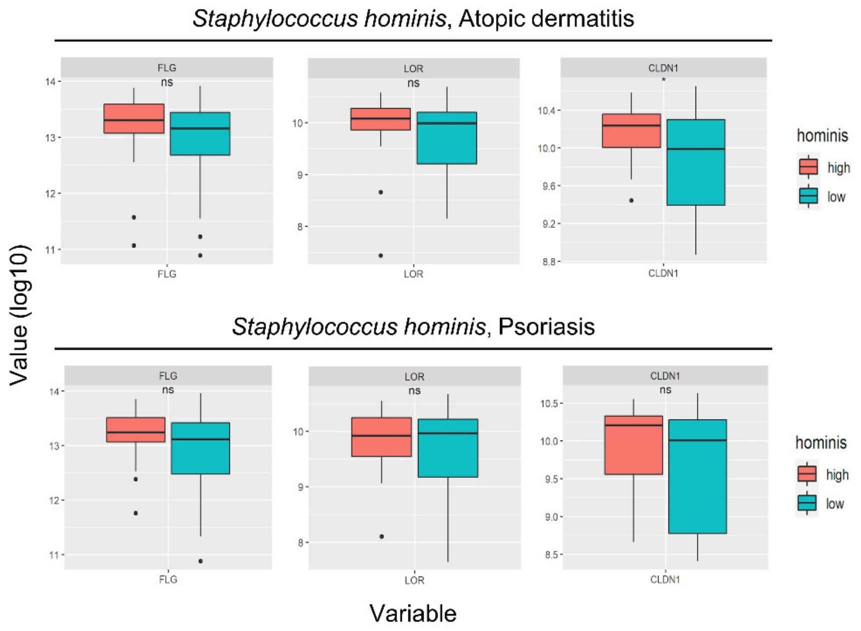

b

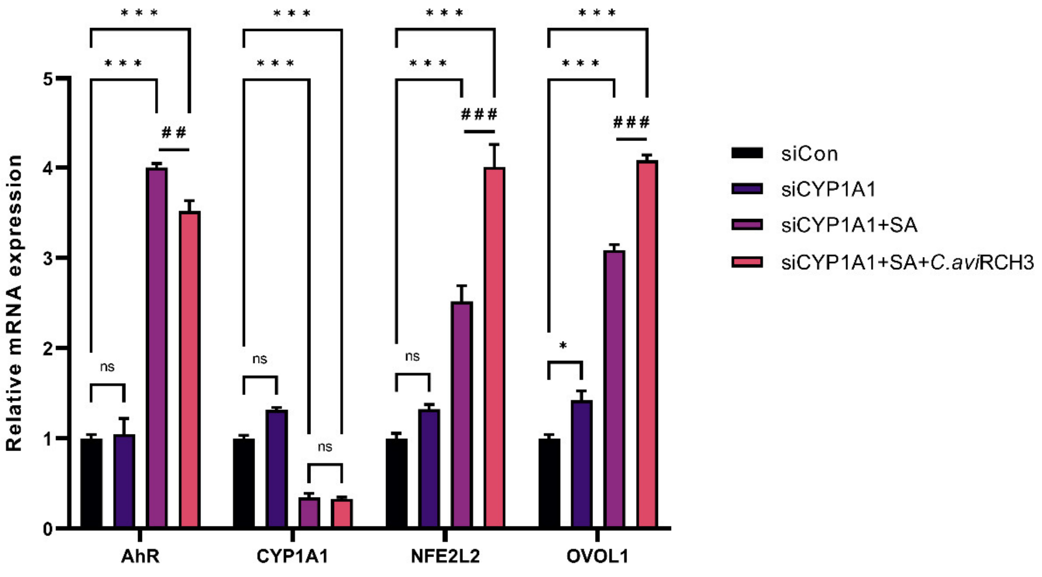

Figure S3. Analysis of AhR/Nrf2 signaling during *CYP1A1* gene silencing after treatment with SA and *C. avidum* G01, and comparison of epidermal terminal factor expression stratified by *S. hominis* abundance. **(a)** Boxplots of gene expression values for *FLG, LOR* and *CLDN1* stratified by abundance of *S. hominis* in AD and Pso cohorts. **(b)** Measurement of mRNA levels of *AHR*, *CYP1A1*, *NFE2L2* and *OVOL1* relative to 18S rRNA in SA treatment and SA with *C. avidum* treatment groups with and without *CYP1A1* gene silencing in HaCaT cells. **p* < 0.05, ****p* < 0.001,

ns: not-significant compared to each si-control group. $^{##}p < 0.01$, $^{###}p < 0.001$, ns: not-significant. $^{##}p < 0.01$, $^{###}p < 0.001$ between SA treatment group and *C. avidum* G01 treatment group after SA treatment in *CYP1A1*-silenced HaCaT cells. Statistical significance was calculated using Bonferroni tests.
